# Understanding the ballistic event: methodology and initial observations

**DOI:** 10.1007/s10853-016-0594-0

**Published:** 2016-11-28

**Authors:** Adam Healey, John Cotton, Stuart Maclachlan, Paul Smith, Julie Yeomans

**Affiliations:** 1 Lucideon Limited, Queens Road, Penkhull, Stoke-on-Trent, Staffordshire ST4 7LQ UK; 2 University of Surrey, Stag Hill Campus, Guildford, Surrey GU2 7XH UK

**Keywords:** Fracture Surface, Boron Carbide, Stereo Imaging, Weibull Modulus, Ballistic Event

## Abstract

The purpose of the study is to accelerate the development of ceramic materials for armour applications by substantially increasing the information obtained from a high-energy projectile impact event. This has been achieved by modifying an existing test configuration to incorporate a block of ballistic gel, attached to the strike face of a ceramic armour system, to capture fragments generated during the ballistic event such that their final positions are maintained. Three different materials, representative of the major classes of ceramics for armour applications, alumina, silicon carbide, and boron carbide, have been tested using this system. Ring-on-ring biaxial disc testing has also been carried out on the same materials. Qualitative analysis of the fracture surfaces using scanning electron microscopy and surface roughness quantification, via stereo imaging, has shown that the fracture surfaces of biaxial fragments and ballistic fragments recovered from the edges of the tile are indistinguishable. Although the alumina and boron carbide fragments generated from areas closer to the point of impact were also similar, the silicon carbide fragments showed an increase in porosity with respect to the fragments from further away and from biaxial testing. This porosity was found to result from the loss of a boron-rich second phase, which was widespread elsewhere in the material, although the relevance of this to ballistic performance needs further investigation. The technique developed in this work will help facilitate such studies.

## Introduction

Ceramic armour material systems have been in use for over one hundred years and since the Vietnam War they have provided protection from high-velocity projectiles to vehicles, aircraft, and personnel on the battlefield. The key property for an armour system is the ability to resist high-energy projectile impacts, which is referred to as ballistic performance. If this can be combined with a low weight (by using low density materials), this offers the prospect of increased fuel economy and/or manoeuvrability. Ideally, these properties will be delivered at a low cost.

Common ceramic materials used for armour systems are aluminium oxide (Al_2_O_3_), known as alumina, silicon carbide (SiC), and boron carbide (B_4_C). Of these, the most widely used is alumina, due to its comparatively low cost of manufacture and effectiveness in protecting against common battlefield threats. Silicon carbide is of lower density and able to resist higher energy impacts but is more expensive. Finally, boron carbide has very low density and high impact resistance, but the high cost often restricts it to applications where weight-saving is critical, such as in aircraft [1]. New ceramic materials are currently in development to improve on these baseline materials.

A significant obstacle in armour development is an incomplete understanding of the phenomena that occur when a high-velocity projectile strikes an armour target. Upon penetration, the bullet and the ceramic strike face undergo a number of processes, such as fragmentation, to dissipate the kinetic energy of the projectile to the extent that what remains is completely stopped by the composite backing of the armour system. The high speed nature (strain rates of approximately 10^8^ s^−1^) and resulting damage to samples inflicted during this interaction, known as the ballistic event, make it difficult to identify the individual mechanisms that dissipate the kinetic energy of an incoming projectile [2, 3]. Further, it is very problematic to systematically alter one property of a ceramic, such as grain size, to gauge its effect on ballistic performance, without inadvertently altering other microstructural parameters. Some properties of ceramics, such as compressive strength, are also known to be strain-rate dependent, causing changes in the material behaviour between test regimes and affecting the nature of brittle fragmentation [4]. Furthermore, the ballistic event is sensitive to changes in strike face and backing material combinations, as well as projectile type, speed, and other variables. Thus, predicting the outcome is challenging [5, 6].

Consequently, the only widely accepted method of assessing how effective a new system or material is at resisting impact is to subject it to ballistic testing. Due to the statistical nature of the mechanical properties of ceramics this is a very expensive process; a robust test requires a minimum of 25 armour samples [7], and over 100 are required for a full understanding of the statistics of the material. This high cost is a significant barrier in the development of new armour materials.

A new test (or suite of tests) that uses economically viable methods to estimate the ballistic performance of a new material is therefore highly sought after. Although not intended to replace the final ballistic evaluation, a preliminary method to screen new candidate armour materials earlier in the development cycle would greatly reduce the cost.

In order to develop a new technique, however, an increased understanding of the ballistic event is required. Currently this information is lacking, in part due to the shortcomings of the ballistic test itself. Armour materials are assessed on a basic pass/fail criterion depending on whether the sample was fully penetrated or not, and little information beyond this is provided (for example, it is not known how close to passing or failing a particular sample was) [8].

Valuable information on the energy dissipation mechanisms that occur during the ballistic event could be obtained from fractographic examination of fragments generated [9, 10], but in a typical experiment the energy of the impact causes fragments to be scattered over a wide range and possibly damaged further post-creation. Researchers have adapted the test to restrain fragments by confining the armour system in a steel box [10, 11], but this has had the effect of altering the stress wave patterns from those that would naturally occur and therefore changed the fragmentation behaviour [12]. Thus, this study had the dual aims of developing a better method to capture fragments and preserve information on the ballistic event, and then studying those fragments to better understand the mechanisms that resulted in their creation. Further, since strain rate throughout a ballistic system decreases with distance from the centre of impact [13], such that fragments obtained from quasi-static test conditions can be compared with ballistic fragments obtained from the edges of the ballistic tile [2], fracture surfaces generated using quasi-static ring-on-ring biaxial disc testing were studied to assess the degree of correlation with captured fragments.

## Materials and methods

### Materials

Three ceramic armour materials were subjected to ballistic testing; Sintox FA alumina (manufactured by Morgan Technical Ceramics), Hexoloy SA silicon carbide (manufactured by Saint-Gobain), and boron carbide (manufactured by 3 M). A summary of the properties relevant to this investigation is given in Table 1; density was obtained using the water displacement method in accordance with BS EN 993-1; Poisson’s ratio and Young’s Modulus were obtained from data sheets from the manufacturers [14–16]; and fracture toughness was obtained from literature for Sintox FA [17], Hexoloy SA [18], and B_4_C [19]. Ballistic performance and cost estimates were provided courtesy of Morgan Advanced Materials.

**Table 1:** Properties of Ballistic Materials [14–19]

Material	Density (g cm^−3^)	Poisson’s ratio	SENB fracture toughness (MPa m^1/2^)	Young’s modulus (GPa)	Ballistic performance	Cost
Sintox FA	3.75	0.23	4.2 ± 0.3	320	Medium	x1
Hexoloy SA	3.13	0.14	2.6 ± 0.1	430	High	x5
3 M B_4_C	2.50	0.18	3.6 ± 0.3	410	High	x10

### Fragment containment

**Figure 1:**
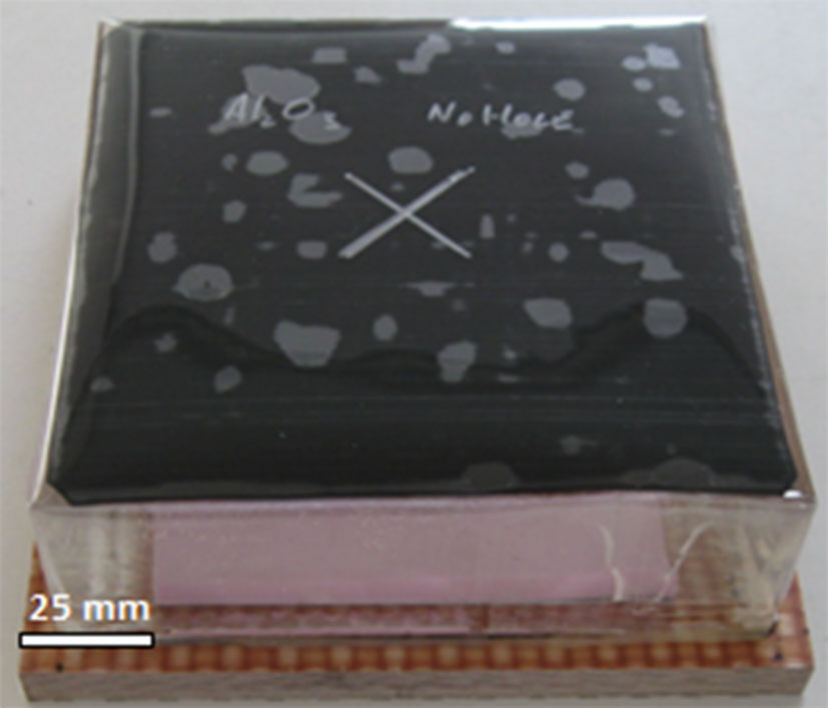
Photograph of Sintox FA ballistic sample with ballistic gel and rubber sheet restraint

Starting from a previous method in which salt was used to capture ejecta from a ballistic event [20], an iterative design process was used to create an improved system. Other restraint methods, including nylon, ballistic film, and salt, were attempted but did not satisfactorily restrain fragments. In the current experiments, a block of ballistic gel was attached to the strike face of the ceramic; in addition to restraining the ejecta, the final positions of fragments were also maintained. Further, comparisons with an unrestrained control sample showed similar fracture patterns, suggesting that interference with the fragment formation was minimal. A 25-mm-diameter hole was cast in the gel to allow unobstructed access of the bullet to the ceramic strike face, and this hole was covered over with a 3-mm-thick rubber sheet to reduce the loss of emitted ejecta. Figure 1 shows the sample set-up for Sintox FA.

### Ballistic testing

Ballistic testing was carried out at the Morgan Composites and Defence Systems facility in Coventry, UK; the projectile was a standard APM2 bullet configured to fire at 1000 m s^−1^. The dimensions of the ceramic tiles were 100 × 100 × 8 mm, and they were bonded to a 150 × 150 × 10 mm glass-fibre composite backing using a proprietary adhesive.

**Figure 2:**
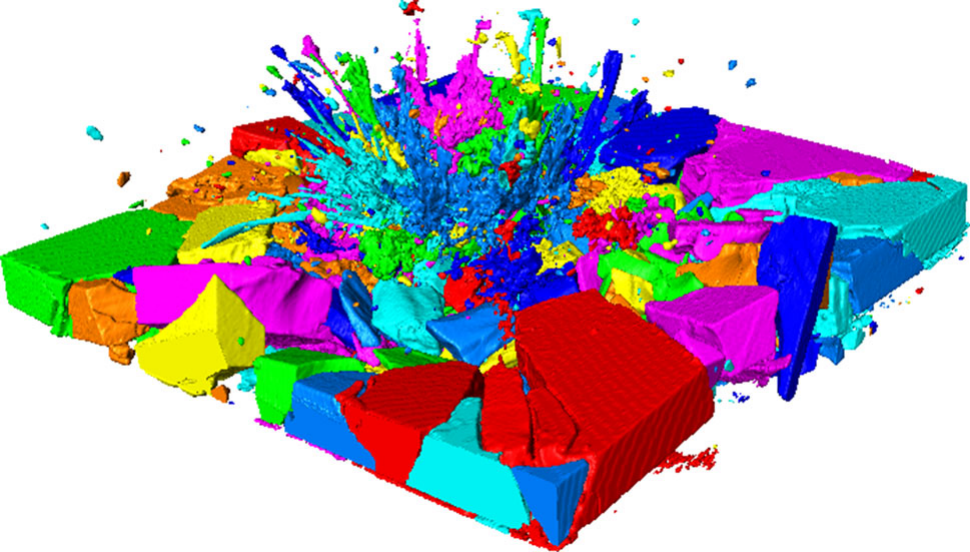
False-colour XCT scan of post-test 3 M B_4_C sample

Post-test quantitative analysis of fragments was attempted non-destructively using X-ray computed tomography (XCT) in partnership with the University of Southampton. A procedure was devised to scan the entire sample at 70 μm resolution then subsequently scan individual areas of interest at 45 μm and 10 μm resolution. A false-colour XCT scan of a post-test sample is shown in Fig. 2.

Qualitative differences between the materials were seen. However, due to the great extent of comminution of fragments, particularly closer to the centre, in many cases, groups of closely packed fragments were recorded as single bodies. This is shown in Fig. 2, where each individually recorded fragment is presented as a distinct colour. In addition, the large difference in densities between the metal bullet, ceramic strike face, gel restraint, and other components made computationally segregating individual materials impossible at smaller scales. Therefore, it was determined that robust analysis could not be carried out using currently available equipment and technology.

Thus, the physical extraction of fragments from the tested samples was necessary, which required a number of steps. After ballistic testing, the system was infiltrated with epoxy resin to promote structural integrity and to maintain the position of fragments during mechanical sectioning. Next, the gel was cut into quarters using a sharp knife, and thin card was inserted into the cuts to prevent them from re-sealing. The remaining materials of the system were subsequently sectioned along the same cut lines using a diamond abrasive saw; extensive damage to the gel was prevented by pre-cutting it, otherwise it would have adhered to the saw blade.

**Figure 3:**
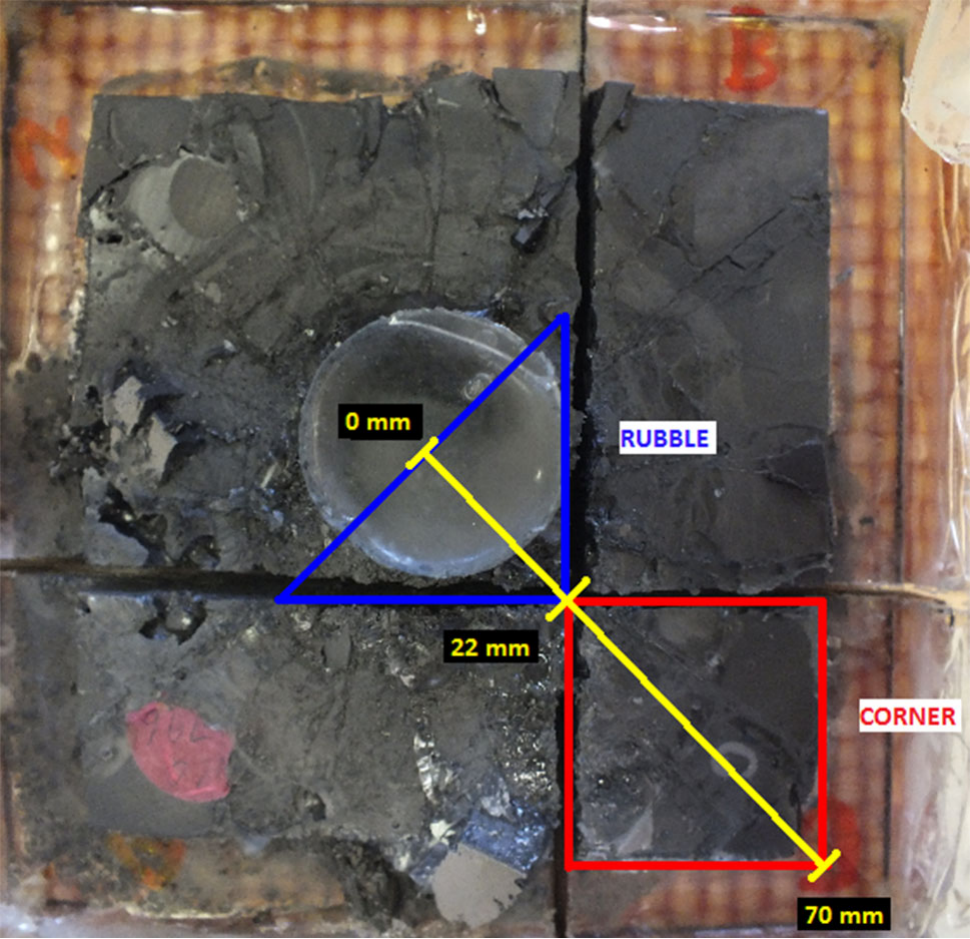
Annotated photograph of post-test Hexoloy SA sample indicating primary areas of fragment extraction and investigation

Figure 3 shows a system sectioned after ballistic testing and designates the primary areas of analysis; the ‘corner’ section (22–70 mm from centre of impact) and ‘rubble’ section (0–22 mm from centre of impact). Fragments were extracted from these discrete sections of the system by burning away the resin. The sections were heated in a furnace set to rise from ambient temperature to 400 °C over 1 h, with a dwell of 2 h then allowed to cool naturally. Once extracted, these fragments were sieved into different size ranges using sieve sizes of 4, 2, 1, 0.5, 0.18, 0.09, 0.063, and 0.045 mm. A magnet was passed over sieved fragments to remove as many steel projectile fragments as possible to prevent them from influencing results.

### Biaxial disc testing

As well as ballistic testing, ring-on-ring biaxial disc testing was carried out on the samples of the same materials. This test was chosen due to the similarities in the deformation with the ballistic test [21, 22] and the large fracture surface area that was generated. It is also a widely available and economical test to carry out, especially when compared with ballistic testing.

Biaxial testing was carried out in accordance with BS ISO 6474-2:2012. Sintox FA and 3 M B_4_C samples were supplied in the form of 36-mm-diameter and 4-mm-thickness discs. The Hexoloy SA samples, however, were supplied in the form of 50 × 50 × 3 mm tiles, and 36-mm-diameter discs were obtained using a coring drill. A total of 15 samples per material were tested.

All samples were sequentially polished to a 1-μm finish on one circular face that was subsequently placed in tension during the test, which was conducted using an Instron 4502 tensile testing machine. It should be noted that having a different surface finish to that of the ballistic tiles potentially adds a further complication when looking for correlations; in this specific case, however, there was no significant difference between results from as-received and polished samples. The loading rig was set up with a 10 kN load cell and a displacement rate of 1.65 mm/min. The ring-on-ring rig itself consisted of a support ring of 30 mm and a loading ring of 12 mm. A rubber disc of 0.5 ± 0.1-mm thickness was placed between the support ring and the sample, and a paper disc was placed between the sample and the loading ring to alleviate friction and account for out-of-planeness. Once fractured, photographs were taken of the sample before each fragment was individually extracted using tweezers and wrapped in aluminium foil to preserve the fracture surfaces.

Fracture surface area was calculated using image analysis software. Crack lengths from both the top view and bottom view were obtained, then the fracture surface area was calculated as a trapezium of height equal to the sample thickness to account for undulations. In addition, a quantitative understanding of the material behaviour can be obtained by calculating the biaxial fracture strength and the Weibull modulus. Biaxial failure strength *σ*_*b*_ was calculated using

1 $$ \sigma_{b} = \frac{{3\left( {1 + \nu } \right)F}}{{2\pi t^{2} }}\left[ {\ln \frac{a}{b} + \frac{{\left( {1 - v} \right)\left( {a^{2} - b^{2} } \right)}}{{\left( {1 + v} \right)2R^{2} }}} \right] $$

where *F* is the failure load; *v* is the Poisson’s ratio; *t* is the sample thickness; *a* is the support ring radius; *b* is the load ring radius; and *R* is the sample radius [23]. A Weibull modulus was also calculated for batches of 15 samples of each material to assess the variability in biaxial strength.

### Fracture surface analysis

Ballistic fragments of ≥0.5 mm and biaxial fragments were ultrasonically washed in deionised water with a surfactant for 30 min to remove loose debris and particulates from the fracture surfaces. Initial fracture surface observations were carried out using a JEOL JSM 6490LV scanning electron microscope (SEM). Surface roughness was quantified using stereo imaging by taking two micrographs at x400 magnification with a eucentric tilt of ±10° and combining them in MeX 5.1 software. Five fragments from each sieve size range were selected, and *R*_a_ and *R*_sm_ results, representing the average line scan height and average line scan periodicity, respectively, were obtained from two sites of analysis per fragment. The chemical composition of the fracture surface of Hexoloy SA was quantified using wavelength dispersive spectroscopy (WDS) at the University of Surrey with a JEOL JSM 7100F SEM combined with a Thermo Scientific MagnaRay spectrometer.

## Results and discussion

### Ballistic testing

**Figure 4:**
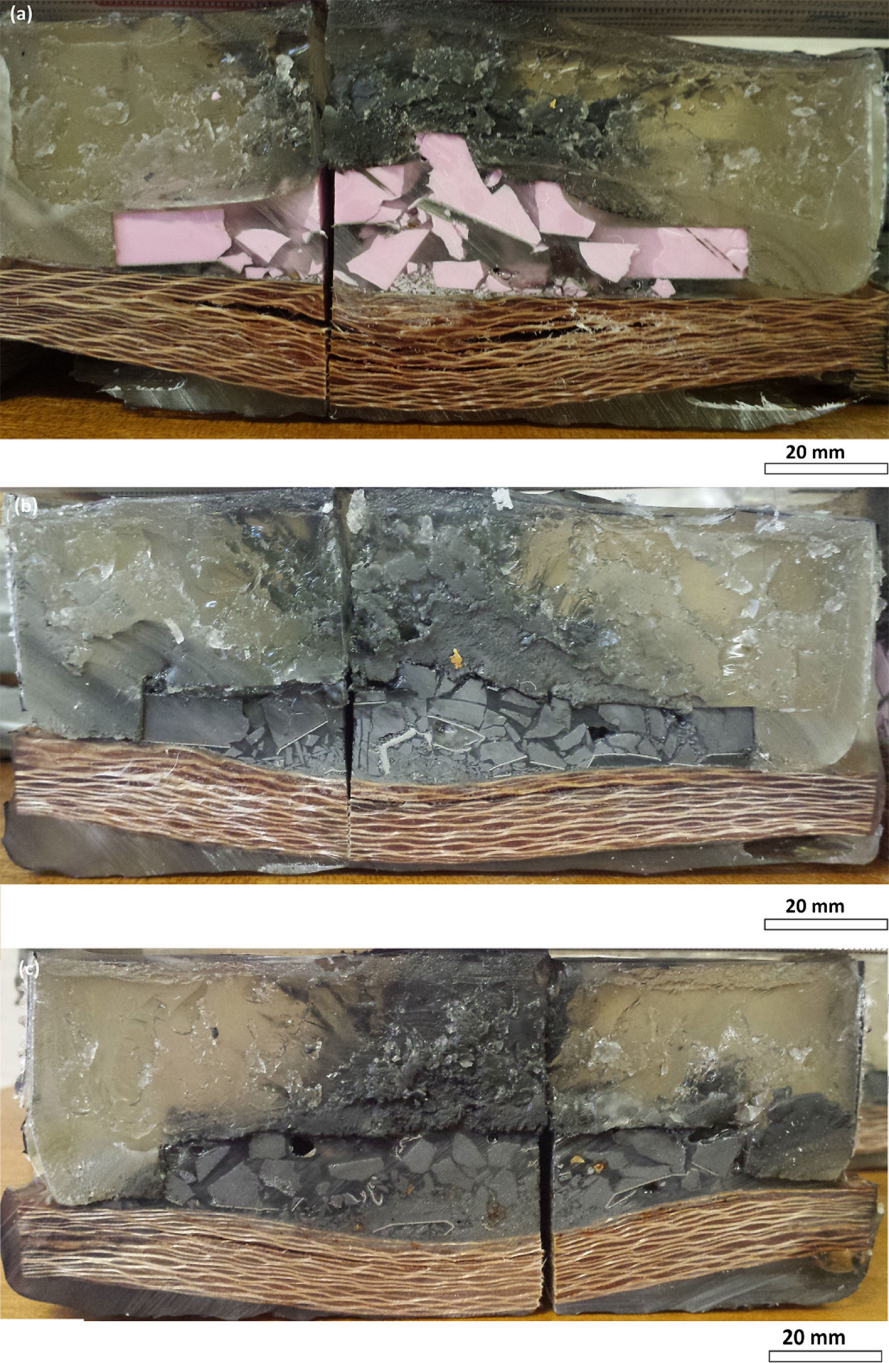
Photographs of off-centre cross sections of ballistic systems *top* Sintox FA; *middle* Hexoloy SA; *bottom* 3 M B_4_C

Fragments were successfully captured from all three materials during ballistic testing. It is notable that the three materials fragmented in different ways when subjected to the same ballistic test; the Sintox fractured into fewer larger fragments, whereas the carbide ceramics exhibited a wider size distribution, as shown in Fig. 4. This suggests that the manner of fragmentation is characteristic of the material rather than the test, which is supported by examples in the literature where it has been reported that an increase in fragment size (and decrease in number of fragments) is related to a decrease in ceramic toughness [3, 10].

**Figure 5:**
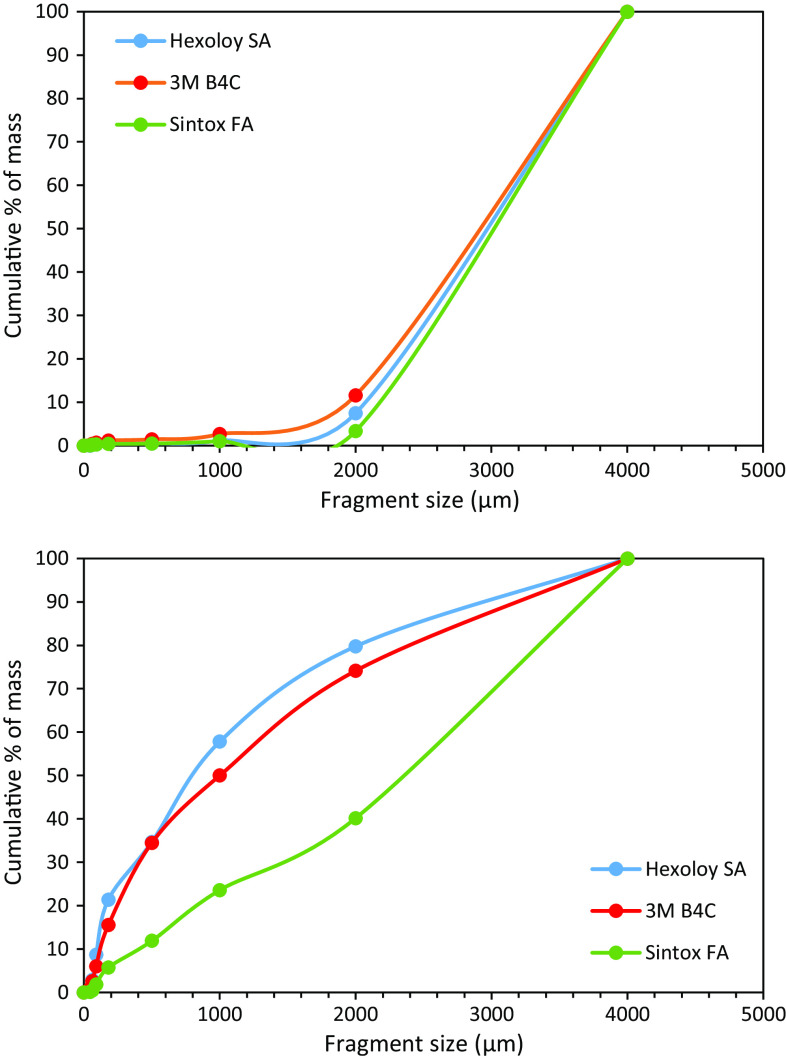
Sieve analysis results for ballistic fragments; *top* corner section; *bottom* rubble section

The total mass of fragments captured by the gel restraint is indicative of the effectiveness of the technique, and was estimated using the mass of fragments extracted from the rubble and corner regions and assuming that the same behaviour was seen in the other quadrants. The total mass values of fragments recovered were 92, 83, and 78% of the intact tiles of Hexoloy SA, 3 M B_4_C, and Sintox FA, respectively, indicating that the majority of the fragments were captured although there were differences in the amounts lost, presumably due to fine particles escaping through the hole made to accommodate the bullet. The size distribution of the captured fragments was obtained from sieve analysis, with the results shown in Fig. 5. The difference in size distributions between rubble and corner sections is expected, as strain rate, which affects fragment size, decreases as distance from centre of impact increases [24]. Whilst all three materials exhibit similar size distributions in the corner section, analysis of the rubble section highlights the differences between them. Sintox FA has approximately 60% of mass contained in fragments ≥2 mm, possibly due to the preferential loss of small particles, whereas 3 M B_4_C and Hexoloy SA have only 20–30 % of mass taken up by ≥2 mm fragments.

### Biaxial disc testing

Table 2 shows the results of biaxial disc testing. The failure stress is lower than typically observed values for other quasi-static fracture tests (e.g., 3-point bending), although this is expected [23] due to the increased size and multi-directional nature of the stress field. Similar to the ballistic results, Sintox FA exhibited the largest fragments and lowest fracture surface area, whereas Hexoloy SA and 3 M B_4_C samples generated larger fracture surface areas. However 3 M B_4_C failed at significantly higher stresses than the Sintox FA, and as the fracture toughness is lower (see Table 1) this implies that the critical flaws are smaller in the boron carbide samples.

**Table 2:** Results for ring-on-ring biaxial disc testing

Material	Failure stress (MPa)	Weibull modulus	Fracture surface area (mm^2^)
Sintox FA	250 ± 30	9 ± 1	700 ± 100
Hexoloy SA	190 ± 20	12 ± 1	1100 ± 100
3 M B_4_C	310 ± 20	17 ± 1	1200 ± 100

The calculated Weibull moduli in Table 2 are higher than data sheet values for Hexoloy SA and 3 M B_4_C, which are 8 and 15, respectively [15, 16]. A data sheet value was unavailable for Sintox. While there needs to be caution when comparing results from different testing conditions, a possible reason for the increased consistency in the results could be that in biaxial flexure the cut edges of the specimens are placed outside of the primary stress field during testing, reducing the influence of cutting damage.

**Figure 6:**
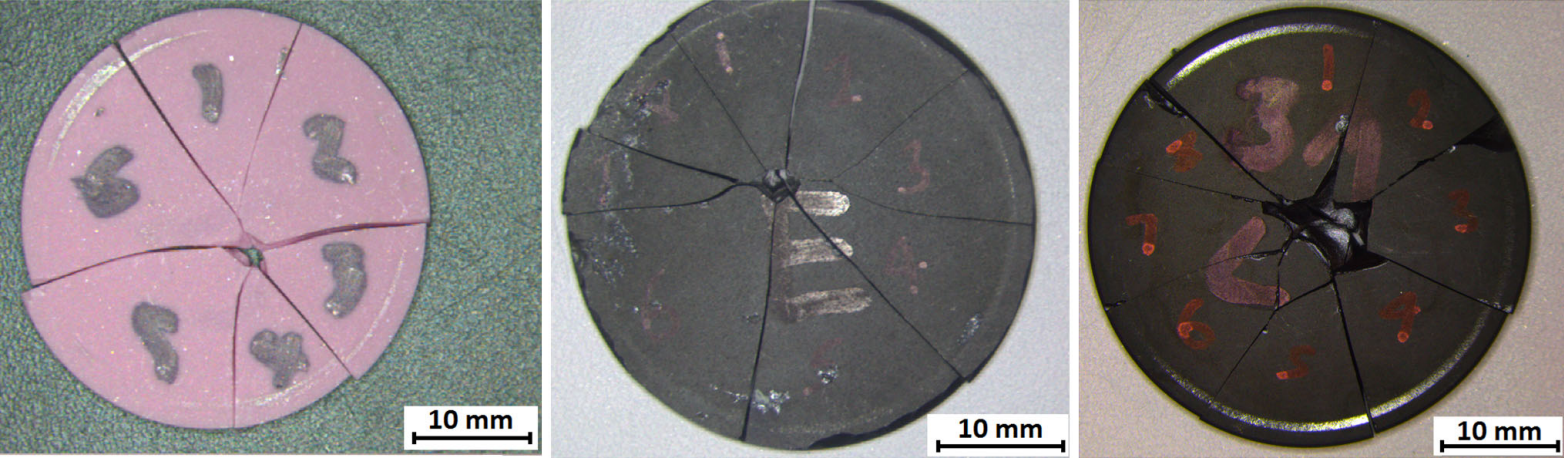
Photographs of samples tested using ring-on-ring biaxial disc testing; *left* Sintox FA; *middle* Hexoloy SA; *right* 3 M B_4_C

Figure 6 shows examples of biaxially fractured samples. As well the differences in fracture surface area, one Sintox FA sample, four Hexoloy SA samples, and all fifteen 3 M B_4_C samples exhibited material loss from the centre of fracture on the compressive side of the sample. This might have occurred as a result of crack bifurcation, known to develop in high-stress samples where the crack energy rises too quickly to be adequately released by a single crack [25]. The extent of this phenomenon could yield additional information about the fracture processes that the material undergoes, potentially providing extra evidence as to how the material fragments form.

## Fracture surface analysis

### Initial observations

**Figure 7:**
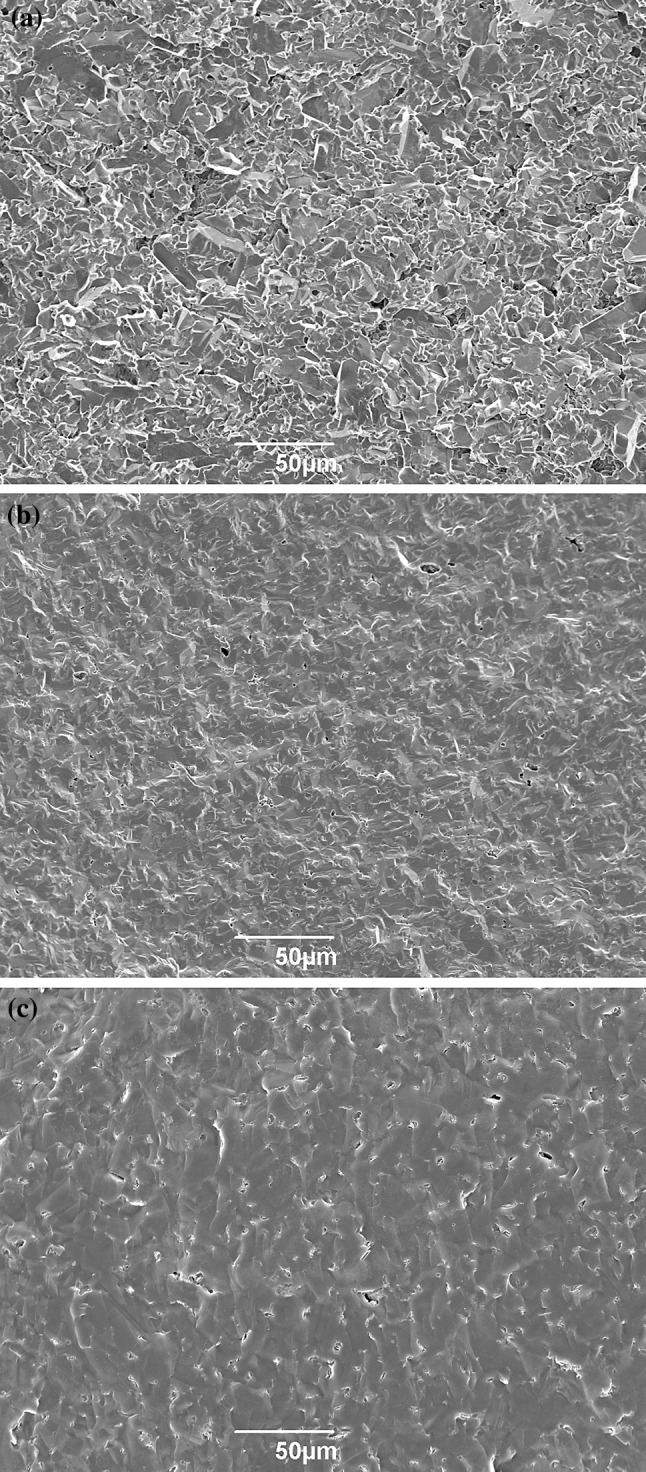
SEM micrographs of typical fracture surfaces; *top* Sintox FA; *middle* Hexoloy SA; *bottom* 3 M B_4_C

Typical micrographs of ballistic fragment fracture surfaces obtained using SEM are shown in Fig. 7. While all three materials show mixed-mode fracture, Sintox FA appears to exhibit primarily intergranular fracture surfaces, whereas Hexoloy SA and 3 M B_4_C show predominantly transgranular fracture surfaces. Such fracture behaviour is typical for these materials [2, 26, 27], and matches previous correlations between fracture toughness and fracture mode in ceramics. For Sintox FA and 3 M B_4_C, there appear to be no noticeable changes in fracture surface behaviour with fragment size or distance from the centre of impact.

**Figure 8:**
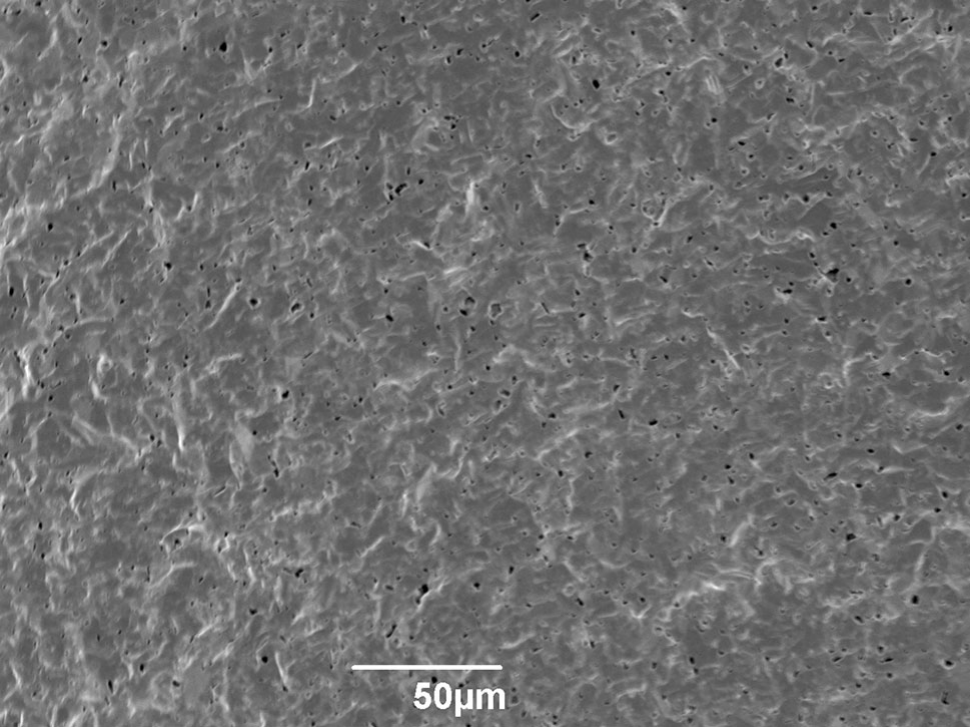
SEM micrograph of typical Hexoloy SA ballistic rubble fragment

However, a notable observation is that in Hexoloy SA, fragments recovered from the rubble (0–22 mm from centre of impact) appear to exhibit a substantial level of porosity on the fracture surface, as shown in Fig. 8, when compared with fragments from the corner (22–70 mm from centre of impact). This is investigated further in a later section.

In comparing biaxial fracture surfaces with ballistic corner fragments, no differences in the appearance of fracture surfaces are immediately apparent for any of the materials. This provides further evidence in support of previous observations that the fracture conditions of material further away from the centre of impact can be recreated under quasi-static conditions. Attempts to locate the fracture origins in the biaxial discs yielded mixed results; while finding fracture origins in Hexoloy SA was relatively straightforward, the Sintox FA microstructure was too obscure to discern any fracture patterns, and material from the centre of 3 M B_4_C, which would have most likely included the fracture origin, was lost during testing.

### Stereo imaging

Table 3 shows the averaged results from the stereo imaging analysis of 3 M B_4_C, Hexoloy SA, and Sintox FA fracture surfaces. The variability within the data is too great to draw firm conclusions, although there appear to be some differences between the materials, with Sintox FA having the roughest (highest *R*_*a*_) fracture surfaces. For each material, the *R*_*a*_ results are similar for the rubble, the corner and the biaxial fracture surfaces, again supporting the potential link between fragments from ballistic and flexural tests. Other researchers have observed a similar lack of difference in fracture surfaces between alumina ceramics tested at different strain rates [28, 29], but this information has not been previously quantified. Caution, however, is required when considering the rubble fragments due to the differences observed in Hexoloy SA fracture surfaces, which are discussed below.

**Table 3:** Stereo imaging surface roughness results of ceramic fragments

Material	Location	Average *R*_a_ (µm)	Average *R*_sm_ (µm)
3 M B_4_C	Rubble	3 ± 1	330 ± 140
	Corner	4 ± 1	410 ± 100
	Biaxial	3 ± 1	270 ± 120
Hexoloy SA	Rubble	3 ± 0	470 ± 160
	Corner	2 ± 1	320 ± 70
	Biaxial	2 ± 1	150 ± 50
Sintox FA	Rubble	5 ± 1	300 ± 40
	Corner	5 ± 1	300 ± 60
	Biaxial	6 ± 2	330 ± 200

### Hexoloy pore analysis

As shown in Fig. 8, Hexoloy SA fragments recovered from the rubble region of the ballistic tile appear to exhibit a significant amount of porosity in the fracture surface when compared with fragments recovered from the corner region. This was confirmed by highlighting the individual pores on micrographs and then by using image analysis software to quantify and compare results, as shown in Table 4; the number of pores found in rubble fragments is significantly higher than in corner fragments. Further, when biaxial fracture surfaces were examined for porosity, the number of pores in biaxial fracture surfaces was found to be similar to that of corner fracture surfaces, further reinforcing links between the two.

**Table 4:** Image analysis results for porosity in Hexoloy SA fracture surfaces

Location	Fragment size (mm)	Average number of pores per 0.1 mm^2^
Rubble	4+	260 ± 70
	4 − 2	890 ± 440
	2 − 1	540 ± 260
	1 − 0.5	400 ± 200
Corner	4+	90 ± 30
	4 − 2	160 ± 60
	2 − 1	290 ± 190
	1 − 0.5	140 ± 80
Biaxial		160 ± 50
Polished		20 ± 10

**Figure 9:**
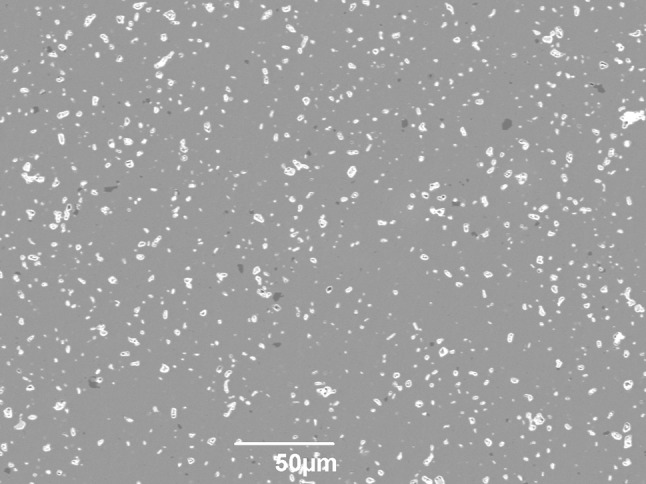
SEM micrograph of polished as-sintered Hexoloy SA

This is a phenomenon that appears to be previously unreported in literature, and so a focussed investigation was warranted. To investigate the possibility that the fracture surface had preferentially formed in response to pre-existing porosity in the Hexoloy SA, an as-sintered sample was polished to 1 µm and analysed using an SEM, as shown in Fig. 9. There appear to be few open pores, although bright regions are seen which are likely to be caused by the rims of pores or inclusions; the brightness of pore edges does not occur in rubble fracture surfaces due to the increased size of pores and the increased contrast caused by surface texture. However, a second phase (darker than the matrix), presumed to result from a boron sintering aid, is seen in the polished sections which is absent from the rubble fracture surfaces.

To investigate this phenomenon further, fragments from the corner and rubble regions were mounted in resin and polished to expose the sub-surface microstructure. They were both found to be identical to as-sintered Hexoloy SA, indicating that this ‘extra’ porosity exists only at fracture surfaces.

Hexoloy SA is known to incorporate boron during manufacture as a sintering aid [30, 31], although the boron is intended to diffuse into the lattice rather than remain, presumably in the form of boron carbide inclusions as seen in the Hexoloy SA micrographs. It is possible that these inclusions may have influenced the fracture path and/or fragmentation behaviour of the Hexoloy SA. Therefore, the presence of second phases in Hexoloy SA fracture surfaces was investigated.

**Figure 10:**
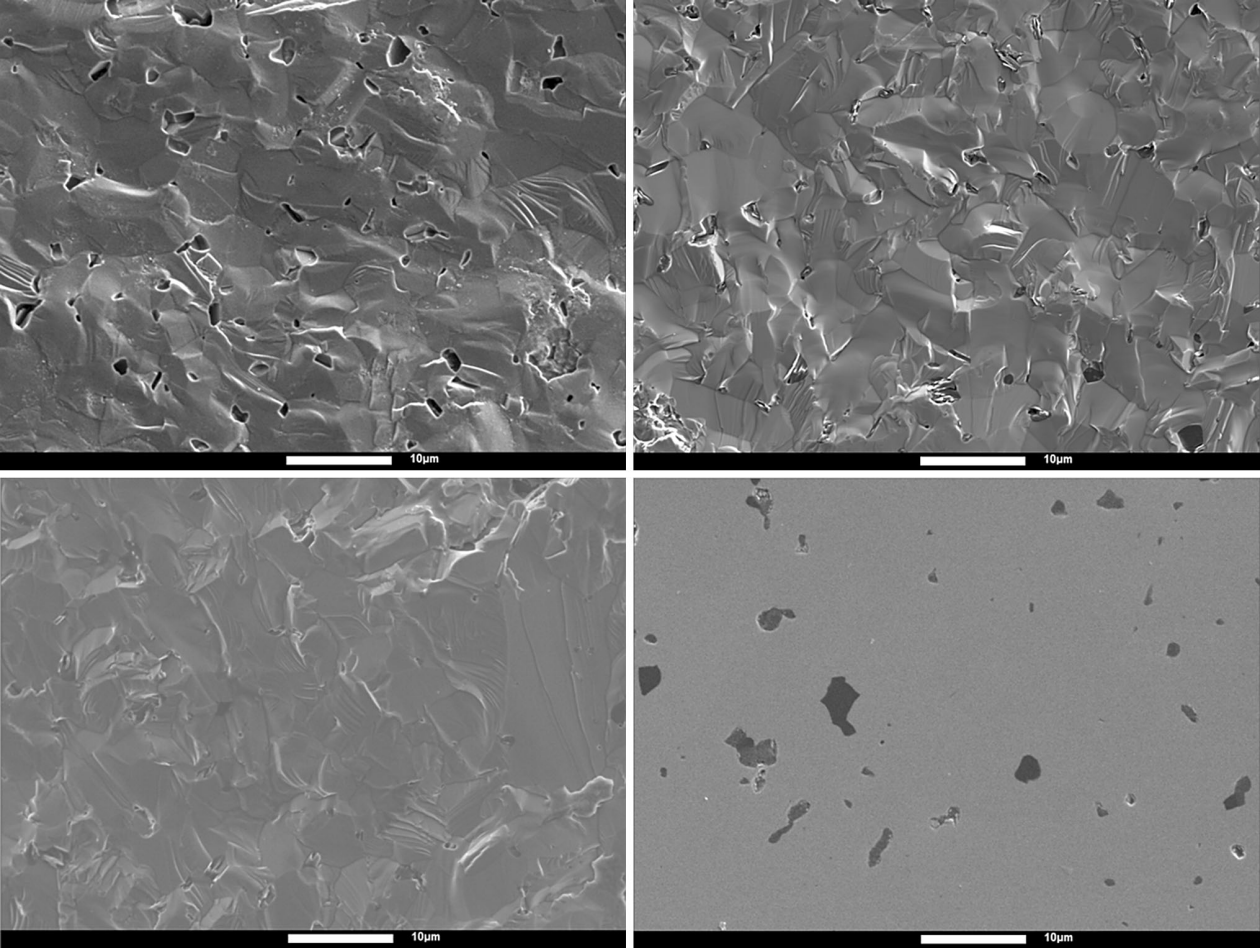
SEM micrographs of Hexoloy SA fracture surfaces; *top-left* rubble; *top-right* corner; *bottom-left* biaxial; *bottom-right* as-sintered

Initial image analysis of SEM micrographs indicated similarities between the total area of pores and defects on the rubble fracture surfaces and the total area of second phase on polished as-sintered material. The links between porosity and second phase were further confirmed by using WDS on rubble, corner, as-sintered, and biaxial Hexoloy SA fracture surfaces, as shown in Fig. 10. The results from WDS are presented in Table 5 and indicate that there is a significant decrease in the atomic percentage of boron observed in rubble fragments when compared with the other samples. Further, the results for corner and biaxial fragments are again similar.

**Table 5:** WDS results for Hexoloy SA fracture surfaces

Location	Atom % B
Rubble	2 ± 1
Corner	8 ± 3
Biaxial	8 ± 3
Polished	10 ± 1

Using the results shown in Table 4 and Table 5, direct comparisons can be made between the increase in porosity and the decrease in boron content, indicating that the porosity is caused by the expulsion of the boron-rich phase during the ballistic event. This phenomenon is more extensive in regions closer to the point of impact, whereas in regions further away the boron count and other facture surface features resemble those obtained by quasi-static biaxial disc testing.

It might be expected that the particles become dislodged because of a change in the local stress state. If the particles are indeed boron carbide (or boron) then they would contract more than the surrounding matrix, since the coefficients of thermal expansion (*α)* are such that $\alpha_{{{\text{B}}_{ 4} {\text{C}}}}$ (and *α*_B_) > *α*_SiC_ and hence the particles would either be debonded from the matrix on cooling from the sintering temperature (which is not evident in the micrographs) or be experiencing radial tension. It would appear that when the stress associated with the ballistic impact is added to this residual stress it is sufficient to dislodge the particles if they are close to the centre of impact but not if they are further away. At this early stage of research, it is unclear as to whether this dislodgement makes a positive, neutral, or detrimental contribution to the ballistic performance of Hexoloy SA. Further work would be needed in order to ascertain whether this phenomenon is more widespread than this single sample of Hexoloy SA, by considering other ceramics with a second phase.

## Concluding remarks

Development of a viable method of cost-effectively assessing the performance of ceramic armour systems is highly sought after; such a technique does not currently exist due to incomplete understanding of the phenomena that occur during a ballistic event. A new protocol has been established to significantly increase the amount of information obtained from current ballistic experiments to inform the development of new tests, and has provided evidence in support of correlations between fragments created at some distance from the centre of impact with those generated in biaxial testing.

By using a block of gel attached to the strike face of ballistic tiles, the fragments generated during the ballistic event were successfully recovered from three different materials. Fractography was carried out on fragments obtained from ballistic and ring-on-ring biaxial tests and surface roughness was quantified using stereo imaging. Using this new technique, the following observations have been made:

- For each of the three materials, ballistic fragments across the size ranges examined were found to exhibit consistency in their appearance and fracture surface.
- Fragments obtained from closer to the edges of the ballistic tile are indistinguishable from fragments created from a biaxial disc test in terms of their fracture surface appearance, roughness, and, in the case of Hexoloy SA, presence of second phase. This suggests that fragments created close to the edges of a ballistic tile can be reliably recreated using biaxial disc testing.
- There is significant porosity in the fracture surfaces of Hexoloy SA fragments recovered from close to the centre of impact. WDS has also indicated a loss of boron when compared with as-sintered Hexoloy SA, suggesting that the pores are caused by loss of a boron-rich phase. This is a previously unreported observation. It remains to be determined what effect, if any, this has on the ballistic performance of Hexoloy SA.

## References

[1] Medvedovski E (2010). Ballistic performance of armour ceramics: influence of design and structure, part I. Ceram Int.

[2] Karandikar PG, Evans G, Wong S, Aghajanian MK (2008). A review of ceramics for armour applications. Ceram Eng Sci Proc.

[3] Woodward RL, Gooch WA, O’Donnell RG, Perciballi WJ, Baxter BJ, Pattie SD (1994). A study of fragmentation in the ballistic impact of ceramics. Int J Impact Eng.

[4] Lankford J (1981). Temperature-strain rate dependence of compressive strength and damage mechanisms in aluminium oxide. J Mat Sci.

[5] Krell A, Strassburger E (2007). Hierarchy of key influences on the ballistic strength of opaque and transparent armour. Ceram Eng Sci Proc.

[6] O’Donnell RG (1993). Deformation energy of Kevlar backing plates for ceramic armours. J Mater Sci Lett.

[7] Kneubuehl BP (1996) Improved test procedure for body armour; statistical base and evaluation program, Proc PASS96, Colchester

[8] Gotts PL, Fenne PM, Leeming DW (2004) The application of critical perforation analysis to UK military and police body armour, Proc PASS04, The Hague

[9] Krell A, Strassburger E (2012). Discrimination of basic influences on the ballistic strength of opaque and transparent ceramics. Ceram Eng Sci Proc.

[10] Wu H, Ghosh S, Dancer C, Todd R (2014). Ballistic damage of alumina ceramics—learning from fragments. Ceram Eng Sci Proc.

[11] Tan ZH, Han X, Zhang W, Luo SH (2010). An investigation on failure mechanisms of ceramic/metal armour subjected to the impact of tungsten projectile. Int J Impact Eng.

[12] Sherman D (2000). Impact failure mechanisms in alumina tiles on finite thickness support and the effect of confinement. Int J Impact Eng.

[13] Ernst CM, Barnouin-Jha OS, Ramesh KT, Swaminathan PK, Kimberley J (2009) Strain rate and dynamic fracturing in planetary-scale impacts. In: 40th lunar and planetary science conference, Texas

[14] Morgan Advanced Materials (2013) Sintox FA Ballistic data sheet. http://www.morgantechnicalceramics.com/media/2328/rugby-sintox-fa-ballistic.pdf. Accessed 19 Aug 2016

[15] Saint-Gobain (2012) Hexoloy SA data sheet. http://www.refractories.saint-gobain.com/hexoloy/hexoloy-sic-materials. Accessed 19 July 2016

[16] 3 M (2015) B_4_C data sheet. http://technical-ceramics.3mdeutschland.de/en/products/3m-ballistic-ceramics.html#c509. Accessed 19 July 2016

[17] Morrell R (2008). Fractography and fracture toughness measurement. Key Engin Mater.

[18] Salem JA (2009). Fracture toughness of thin plates by double-torsion test method. Ceram Engin Sci Proc.

[19] Thévenot F (1990). Boron carbide—a comprehensive review. J Euro Ceram Soc.

[20] Kecskes LJ, Magness LS (2007) Recovery apparatus for fragmented ballistic materials and method for collection of the same, Patent number US 7,163,205, Washington, DC

[21] Strassburger E, Hunzinger M, Patel P, McCauley JW (2013). Analysis of the fragmentation of AlON and spinel under ballistic impact. J Appl Mech.

[22] Wereszcak A, Swab JJ, Kraft RH (2005). Effects of machining on the uniaxial and equibiaxial flexure strength of CAP3 AD-995 Al2O3.

[23] Morrell R (2007) Measurement good practise Guide No.12—biaxial flexural strength testing of ceramic materials, NPL Guide

[24] Lankford J, Blanchard CR (1991). Fragmentation of brittle materials at high rates of loading. J Mater Sci.

[25] Hull D (1999). Fractography: observing, measuring and interpreting fracture surface topography.

[26] MoberlyChan WJ, Cao JJ, Gilbert CJ, Ritchie RO, De Jonghe LC (1998) The cubic-to-hexagonal transformation to toughen SiC. Ceramic Microstructures, pp. 177–190

[27] Horsfall I, Edwards MR, Hallas MJ (2010) Ballistic and physical properties of highly fractured alumina, Staff Publications—Cranfield Defence and Security, London

[28] Gàlvez F, Rodríguez J, Gàlvez VS (1997). Influence of the strain rate on the tensile strength in aluminas of different purity. J Phys IV.

[29] Belenky A, Rittel D (2012). Static and dynamic flexural strength of 99.5% alumina: relation to surface roughness. Mech Mater.

[30] Ischenko V, Jang YS, Kormann M, Greil P, Popovska N, Zollfrank C, Woltersdorf J (2011). The effect of SiC substrate microstructure and impurities on the phase formation in carbide-derived carbon. Carbon.

[31] Elliot S (2007). Silicon carbide ceramic armour. AM&P Mag.

